# Dyadic Coping in Patients Undergoing Radiotherapy for Head and Neck Cancer and Their Spouses

**DOI:** 10.3389/fpsyg.2018.01780

**Published:** 2018-10-15

**Authors:** Hoda Badr, Krista Herbert, Mark D. Bonnen, Joshua A. Asper, Timothy Wagner

**Affiliations:** ^1^Department of Medicine, Baylor College of Medicine, Houston, TX, United States; ^2^Department of Psychology, Rowan University, Glassboro, NJ, United States; ^3^Department of Radiation Oncology, Baylor College of Medicine, Houston, TX, United States

**Keywords:** head and neck cancer, couples, caregiving, dyadic coping, radiotherapy, psychosocial intervention, depression, dyadic adjustment

## Abstract

**Background:** Head and neck cancer (HNC) adversely affects the psychological (i.e., depression, anxiety) and marital adjustment of patients and their spouses. Dyadic coping refers to how couples cope with stress. It includes positive actions like sharing practical or emotional concerns (i.e., problem- and emotion-focused stress communication; PFSC, EFSC), and engaging in problem- or emotion-focused actions to support each other (problem- and emotion-focused dyadic coping; PFDC, EFDC). It also includes negative actions like avoidance (negative dyadic coping; NEGDC). In this secondary analysis of a randomized pilot trial of a couple-based intervention called SHARE (Spouses coping with the Head And neck Radiation Experience), we first examined associations between patients’ and spouses’ dyadic coping (and satisfaction with dyadic coping; SATDC) and their own/each other’s psychological and marital adjustment. Next, we examined the effects of SHARE relative to usual medical care (UMC) on patients’ and spouses’ dyadic coping. Finally, we examined whether changes in dyadic coping were associated with changes in patients’ and spouses’ psychological and marital adjustment.

**Methods and Measures:** Thirty HNC patients (80% men) and their spouses (*N* = 60) completed baseline surveys prior to initiating radiotherapy (RT) and were randomized to SHARE or UMC. One month after RT, they completed follow-up surveys.

**Results:** Baseline multilevel Actor-Partner Interdependence Models revealed significant actor effects of PFSC (effect size *r* = −0.32) and PFDC (*r* = −0.29) on depression. For marital adjustment, significant actor effects were found for PFSC, PFDC, EFDC, and SATDC (*p* < 0.05, *r* = 0.23 to 0.38). Actor (*r* = −0.35) and partner effects (*r* = −0.27) for NEGDC were also significant. Moderate to large effect sizes were found in favor of SHARE on PFSC (Cohen’s *d* = 1.14), PFDC (*d* = 0.64), NEGDC (*d* = −0.68), and SATDC (*d* = 1.03). Improvements in PFDC were associated with reductions in depression and anxiety (*p* < 0.05); and, improvements in SATDC were associated with improvements in anxiety and marital adjustment (*p* < 0.05).

**Conclusion:** The SHARE intervention improved positive and decreased negative dyadic coping for patients and spouses. Increases in positive dyadic coping were also associated with improvements in psychological and marital adjustment. Although findings are preliminary, more research on ways to integrate dyadic coping into oncology supportive care interventions appears warranted.

## Introduction

Head and neck cancers (HNCs) are malignancies of the larynx, pharynx, nasopharynx, and oral cavity. They account for approximately 560,000 new cases worldwide and 3% of cancer cases in the United States (Siegel et al., 2017). An analysis of Surveillance, Epidemiology, and End Results (SEER) data found that being married reduced the risk of dying from HNC by 33% (hazard ratio [HR] = 0.67; Aizer et al., 2013). Married patients also have significantly better performance status scores during HNC treatment than their unmarried counterparts (Konski et al., 2006). One possibility is that spouses (i.e., husbands/wives or significant others) often serve as primary caregivers and provide support and care to facilitate patient adherence to medical recommendations (Family Caregiver Alliance [FCA], 2012).

Radiation therapy (RT) is a common treatment modality for the management of HNC. Patients undergo RT for 5 days a week for 6–7 weeks. RT is either administered alone or it is combined with other treatments (e.g., chemotherapy or surgery; National Comprehensive Cancer Network [NCCN], 2013). Given the dosage of radiation required to successfully treat HNC tumors and the sensitivity of the location that is targeted, patients experience side effects (e.g., mucositis, xerostomia) and functional challenges (e.g., dysphagia) that make eating, drinking, and communicating extremely difficult (Epstein et al., 2001). Their quality of life (QOL) is also adversely affected due to psychological distress, rapid weight loss, dehydration, and malnutrition (Epstein et al., 2001; Massie, 2004; Duffy et al., 2006). Psychoeducational interventions can improve QOL and enhance coping with cancer (Faller et al., 2013); however, few programs have been developed specifically for HNC patients (Semple et al., 2013).

Even though being married can be beneficial for HNC patients, cancer exacts a heavy toll on spouses. Spouses serving in a caregiving role experience higher rates of distress (i.e., anxiety and depression), weakened immune responses, a greater likelihood of long-term medical problems, and higher mortality rates than their non-caregiving counterparts (Applebaum and Breitbart, 2013). In HNC, spouse distress rates are comparable to or higher than those of patients (Hodges et al., 2005; Verdonck-de Leeuw et al., 2007). Spouses also report unmet needs during the critical period when patients are undergoing RT including help with balancing competing roles/responsibilities, making time for self-care, and finding effective strategies for encouraging patient self-management (Badr et al., 2016). Addressing spouse distress is important in its own right. Moreover, distressed spouses may be unable to provide adequate caregiving and support to the patient during RT.

Cancer also challenges couples’ established communication patterns, roles, and responsibilities (Manne and Badr, 2010). Whereas some individuals report that cancer improved their relationships, others experience adjustment and communication challenges that fuel interpersonal conflict and can even lead to divorce (Karraker and Latham, 2015; Badr et al., 2016). In HNC, declines in marital adjustment have been reported 1 year after treatment (Gritz et al., 1999). This is concerning because the quality of marital interaction is related to both psychological adaptation and health outcomes in cancer (Burman and Margolin, 1992).

One aspect of cancer that may be particularly challenging to negotiate for couples coping with HNC is self-care/self-management during RT. Self-management refers to daily activities that minimize the impact of illness on functioning and well-being (Clark et al., 1991). HNC involves considerable self-management during and after RT. For example, patients are instructed to significantly alter their diets to prevent malnutrition, sip or spray the mouth regularly with water to prevent dehydration, use salt-soda rinses 8–10 times a day and saliva substitutes to control xerostomia, practice multiple daily repetitions of exercises to facilitate return to a normal swallow, and engage in intensive oral care routines to control mucositis and prevent dental carries (Jansma et al., 1992; Nguyen et al., 2007). Unfortunately, rates of non-adherence are high – up to 72% of patients are non-adherent with oral care recommendations and 87% are non-adherent with swallowing exercises (Epstein et al., 1995; Shinn et al., 2013). From a medical perspective, poorly managed side effects can lead to treatment interruptions, social/emotional problems, and a more complicated and costly rehabilitation process (Nitenberg and Raynard, 2000; Trotti et al., 2003). From a relationship perspective, patient non-adherence sets the stage for power struggles and conflict between the patient and caregiving spouse (McGuire, 2003). Indeed, a recent study found that 83% of HNC spouses reported increased marital conflict during RT (Badr et al., 2016). Another found that 54% of 125 HNC couples identified side effect management as a topic of considerable concern or contention (Badr et al., 2015). Thus, in order to maximize QOL and health outcomes, it is imperative to address both patient and spouse self-management as well as how the couple relates to one another and coordinates care and support during this critical period.

### The SHARE Intervention

Based on the above, we developed a 6 week telephone-based intervention called SHARE (Spouses coping with the Head And neck Radiation Experience). SHARE actively involves patients and their spouses by: (1) educating both partners about acute and long term side-effects and side-effect management; (2) teaching strategies to improve dyadic coping; and (3) teaching self-management skills that are tailored to each partner’s role (as patient or spouse). By teaching couples the skills to coordinate self-management and support at the start of RT, a major goal is to fortify the couple unit and mitigate the potentially adverse effects of RT on both partners’ psychological and marital adjustment.

### Theoretical Basis

The SHARE intervention is grounded in self-regulation theory (Cameron and Leventhal, 2012) and Bodenmann’s Systemic-Transactional Model (STM) of coping with stress (Bodenmann, 2005). Self-regulation theory posits that goal-setting and self-monitoring improve self-management. However, gaps exist in our understanding of how patients and partners balance autonomy and support, and how they coordinate care while under stress. The STM posits a model of dyadic coping whereby relational partners try to mitigate the adverse impact of stress that affects either one or both partners. Dyadic coping is process consisting of (1) the communication of problem- or emotion-focused stress by partner A, (2) the awareness or perception of partner A’s stress by partner B, and (3) partner B’s coping reaction to partner A’s behavior. Dyadic coping includes positive actions like sharing practical or emotional concerns (i.e., problem- and emotion-focused stress communication), supportive actions like helping a partner to engage in positive reframing and problem-solving (problem-focused dyadic coping), and offering empathic understanding (emotion-focused dyadic coping). It also includes negative actions like distancing, blaming, or minimizing the seriousness of a partner’s stress (negative dyadic coping). Whereas positive dyadic coping is important for helping couples resolve problems and reduce emotional arousal, negative dyadic coping is considered a maladaptive couples’ coping strategy. Finally, satisfaction with dyadic coping refers to each partner’s view of how they cope as a couple. Despite research examining dyadic coping in a variety of illness contexts, very little is known about the dyadic coping process in HNC or how one partner’s dyadic coping affects the other partner.

Our interest in dyadic coping in the context of RT for HNC stemmed from the idea that illness-specific coping efforts are often most effective at producing positive outcomes when both partners collaborate in illness management (Kuijer et al., 2000; Berg and Upchurch, 2007). Although findings regarding the association between the different forms of dyadic coping and psychological adjustment have been mixed (Badr et al., 2010; Meier et al., 2011; Rottmann et al., 2015), research has consistently demonstrated significant associations with marital adjustment (Falconier et al., 2015; Traa et al., 2015). STM interventions have also resulted in improvements in both psychological and marital adjustment in couples coping with breast and gynecological cancers (Kayser et al., 2010; Heinrichs et al., 2011). Couples who take a dyadic approach to dealing with the challenges of RT from the initiation of treatment may thus benefit in terms of better symptom control and adjustment for the patient, better psychological adjustment for the spouse, and better marital adjustment for the couple.

### The Current Study

In a pilot randomized controlled trial, significant treatment effects (medium in magnitude) were observed for SHARE relative to UMC with regard to HNC-specific physical symptom burden (Cohen’s *d* = −0.89) and symptom interference (*d* = −0.86). Medium-to-large effects favoring SHARE were also found for patient and spouse depressive symptoms (*d* = −0.84) and cancer-specific distress (*d* = −1.05) (Badr et al., unpublished). However, this global analysis did not allow for an examination of whether the dyadic coping skills taught in the intervention were related to improvements in patient and spouse psychological and marital adjustment. Therefore, this paper reports on a secondary analysis that was conducted to examine: (1) associations between patient and spouse reports of their own positive and negative dyadic coping efforts at baseline (prior to randomization) and their own and each other’s psychological and marital adjustment; (2) effects of the SHARE intervention on patient and partner engagement in positive and negative dyadic coping relative to usual medical care (UMC); and (3) whether changes in positive and negative dyadic coping are associated with changes in psychological and marital adjustment. Based on the STM and previous research, we hypothesized that engaging in more positive and less negative dyadic coping would be associated with better psychological (i.e., fewer symptoms of depression and anxiety) and marital adjustment for both the person engaging in dyadic coping and that person’s partner. We also hypothesized that patients and spouses who received the SHARE intervention would show greater improvements in positive dyadic coping (and greater reductions in negative dyadic coping) than those receiving UMC. Finally, we expected that improvements in positive dyadic coping and reductions in negative dyadic coping from baseline to 1-month follow up would be associated with improvements in psychological and marital adjustment for both HNC patients and their spouses.

## Materials and Methods

### Procedures

The study was reviewed and approved by the Baylor College of Medicine Institutional Review Board. Patients were eligible if they (1) were initiating radiation treatment for HNC; (2) were spending more than 50% of the time out of bed on a daily basis, as measured by an Eastern Cooperative Oncology Group (ECOG) performance status of < 2; and (3) had a spouse/partner who lived with them. In addition, patients and caregivers had to: (4) be >18 years old; (5) have the ability to speak/read English; and (6) be able to provide informed consent. Patients were identified through medical chart review and approached to participate during a pre-treatment clinic visit. If spouses were not present, permission was obtained to contact them by phone. All eligible couples who were approached were asked to complete a one-page anonymous survey that asked about their health (i.e., NCCN distress thermometer, items from the MD Anderson Symptom Inventory; MDASI) and socio-demographic characteristics regardless of whether they agreed to participate. Patients and spouses who provided written informed consent separately completed a baseline survey and either returned it by mail or at their next clinic visit. Couples who returned the questionnaire were randomly assigned to either the 6-week SHARE intervention or UMC. Couples in both conditions completed follow-up paper-and-pencil surveys 1 month after RT and received gift cards upon return of each completed survey ($10 for baseline and $20 for the one-month follow-up).

### Measures

#### Dyadic Coping

The 37-item Dyadic Coping Inventory (DCI; range 1 = very rarely to 5 = very often) assesses stress communication and dyadic coping (Bodenmann, 2008; Randall et al., 2016). Given concerns about participant burden, only 11 items from the DCI assessing stress communication, supportive and negative dyadic coping, and satisfaction with dyadic coping were used. Patients and spouses rated how they communicate when they are feeling stressed because of cancer on a 5-point Likert-type scale (1 = very rarely to 5 = very often). The specific items/sub-scales are below. Mean scores for individual subscales were used in the analysis.

##### Stress communication

Two items assessed problem-focused stress communication (PFSC; e.g., *I let my partner know that I appreciate his/her practical support, advice, or help*), and two items assessed emotion-focused stress communication (EFSC; e.g., *I tell my partner openly how I feel and that I would appreciate his/her support*). In this study, internal consistency reliability (Cronbach’s alpha) for PFSC was α_patients_ = 0.62 and α_spouses_ = 0.65, and for EFSC it was α_patients_ = 0.60 and α_spouses_ = 0.58.

##### Supportive dyadic coping

Two items assessed problem-focused dyadic coping (PFDC; e.g., *I help my partner to see the situation in a different light*) and three items assessed emotion-focused dyadic coping (EFDC; e.g., *I show empathy and understanding to my partner*). Internal consistency reliability (Cronbach’s alpha) for PFDC was α_patients_ = 0.60 and α_spouses_ = 0.66, and for EFDC it was α_patients_ = 0.75 and α_spouses_ = 0.84.

##### Negative dyadic coping

Two items assessed negative dyadic coping (NEGDC; i.e., *When my partner is stressed, I tend to withdraw* and, *I blame my partner for not coping well enough with stress*). Internal consistency reliability was α_patients_ = 0.66 and α_spouses_ = 0.62.

##### Satisfaction with dyadic coping

A single item assessed satisfaction with dyadic coping (SATDC; i.e., *I am satisfied with the support I receive from my partner and the way we deal with cancer related stress together*).

#### Psychological Adjustment

Both depression and anxiety symptoms were assessed. The 6-item PROMIS short-form depression measure assesses negative mood and views of the self over the past 7 days (Pilkonis et al., 2011). Sample items are, “I felt unhappy” and “I felt worthless.” The 6-item PROMIS short-form anxiety measure assesses fear, anxious misery (e.g., worry), and hyperarousal over the same time-frame (Pilkonis et al., 2011). For both measures, responses range from 1 (never) to 5 (always) and are summed to form a raw score that can then be rescaled into a T-score (standardized) with a mean of 50 and standard deviation (SD) of 10 using tables from the PROMIS website. In this study, internal consistency reliability (Cronbach’s alpha) for depression was α_patients_ = 0.83 and α_spouses_ = 0.90, and for anxiety it was α_patients_ = 0.92 and α_spouses_ = 0.90.

#### Marital Adjustment

The 7-item, short version of the Dyadic Adjustment Scale (DAS-7) has been found to conserve, without loss of variance, the pattern of relations found between the longer, 32-item DAS and related constructs (Hunsley et al., 2001). Three items ask subjects to report on the extent of agreement/disagreement between partners on various issues (e.g., “time spent together); items are rated from 0 = always disagree to 6 = always agree. Three items ask how often various events occur between partners (e.g., “have a stimulating exchange of ideas); items are rated from 0 = never to 5 = more often than once a day. Finally, one item asks about the overall degree of happiness in the relationship (0 = extremely unhappy to 6 = perfect). Items are summed to create a total score ranging from 0 to 36; scores less than 21 indicate marital distress. Internal consistency reliability (Cronbach’s alpha) was 0.85 for spouses and 0.74 for patients.

#### Demographic and Medical Variables

Patients and spouses reported their age, ethnicity, race, education level, employment status, marital status, and length of relationship. Patients also reported on time since initial diagnosis, disease stage, and comorbidities. Where possible, this data was verified by the patient’s electronic medical record.

#### Study Conditions

##### UMC

UMC consisted of standard oncologic care for the patient (e.g., routine management of physical and psychological symptoms, and basic discussions about prognosis/treatment side effects). Partners were welcome to attend patients’ routine clinic and treatment visits but were not required to do so.

##### SHARE

In addition to UMC, patients and spouses each received a manual with units covering: (1) self-care, (2) symptom management, (3) stress management, (4) coping with cancer as a team, (5) managing post-treatment recovery together, and (6) finding the new normal together after cancer. Units 1–3 focused on individual skills, with tailoring based on role. For example, patient-specific content included self-care, soliciting support, and balancing accepting help with autonomy. Spouse-specific content included caregiver self-care, caregiving skills (e.g., hygiene care, meal preparation, identifying red flag symptoms), and strategies for supporting patient self-management. Units 4–6 were dyadic in focus, so manual content was the same for both partners.

In addition to the tailored manuals, patients, and spouses each received an educational CD and DVD that reinforced covered materials (e.g., relaxation and swallowing exercises), and six telephone-sessions corresponding to the units in the manual with an interventionist who had Masters’-level training in mental-health counseling (60-min each). We opted for telephone as opposed to in-person delivery due to research citing low attendance as a barrier to clinic-based program delivery (Ostroff et al., 2004), and research suggesting that telephone delivery is convenient, personal, and preferable to other home-based formats (i.e., videophone, Skype) given the body image and social withdrawal issues that are documented in HNC (Katz et al., 2002).

Intervention sessions were digitally audio-recorded to ensure fidelity. During the sessions, interventionists reviewed homework and manual content, guided participants through in-session activities, and assigned/reviewed homework to reinforce practice of skills taught. Patients and spouses each received separate calls for units 1, 2, and 3. The purpose was threefold: (1) to facilitate rapport between the interventionist and individual members of the couple before moving to the joint sessions; (2) to allow more in-depth coverage of tailored materials; and (3) to provide more time for patients/spouses to practice individual skills and receive feedback before moving to learning dyadic skills. Couples participated in joint calls (sessions 4, 5, and 6) with the interventionist via speaker-phone or three-way call.

The timeline for session delivery was based on the known symptom burden and recovery process for patients undergoing RT for HNC. Because early intervention has been shown to improve treatment tolerance and outcomes (Paccagnella et al., 2010), we delivered the first 4 sessions on a weekly basis starting the first week of RT. The goal was to teach self-care and coping skills before severe symptom onset (which usually occurs during week 4 or 5 of RT). A 4-week break followed to allow time to apply the skills learned and for patients to recuperate. The last 2 sessions were scheduled following the 4-week break due to their focus on managing long-term side effects and the transition to survivorship.

#### Data Analysis

Descriptive statistics (e.g., means, standard deviations) were calculated for each of the major study variables, and paired *t*-tests were conducted to determine whether mean scores differed for patients and spouses at baseline. Associations between the study outcomes and medical (i.e., number of comorbidities, length of time since initial diagnosis, stage at diagnosis (i.e., stage 4 vs. stages 1, 2, and 3) and socio-demographic variables (i.e., age, length of relationship, race/ethnicity [i.e., Anglo/white vs. other], employment status [employed full/part time vs. unemployed/retired]) were examined using Pearson’s correlations for continuous variables and Analyses of Variance (ANOVAs) or *t*-tests for the categorical/dichotomous variables to determine potential model covariates. Of all the medical and socio-demographic variables that we examined, only age and length of relationship were significantly associated with the study outcomes (*p* < 0.05). However, age and length of relationship were highly significantly correlated (*r* = 0.73). Given the small sample size and desire to conserve degrees of freedom, we opted to only include age as a covariate. Moreover, age was significantly correlated with all 3 study outcomes and length of relationship was only significantly correlated with anxiety.

Because data from married couples tend to be related, analyses must adjust for this non-independence so that statistical significance tests are not biased, and model the interdependence or mutual influence process itself. The Actor-Partner Interdependence Model (APIM) accomplishes both goals by utilizing a multilevel modeling approach in which data from two dyad members are treated as nested scores within the same group (Kenny et al., 2006). The APIM suggests that a person’s independent variable score affects his or her own dependent variable score (known as the actor effect), and his or her partner’s dependent variable score (known as the partner, or cross-spouse effect). We can also determine whether these effects differ depending on role (i.e., whether the actor is a patient or spouse) or gender (i.e., whether the actor is a man or a woman). Because it is not clear whether gender or role is a stronger predictor of adjustment to cancer and because the majority of dyads in this small sample study comprised male patients and female spouses, we chose to focus on role effects.

A series of APIM analyses were conducted to examine the baseline actor and partner associations for each of the dyadic coping predictor variables of interest (i.e., problem- and emotion-focused stress communication, problem- and emotion-focused dyadic coping, negative dyadic coping, and satisfaction with dyadic coping) and the study outcomes (i.e., depression, anxiety, and marital adjustment), controlling for participant age. We also tested whether the association between a specific dyadic coping behavior and the outcome of interest differed depending on role (1 = patients and −1 = spouses). The continuous predictor variables were standardized, and the error terms were allowed to differ for the two dyad members. Partial correlations (r) were used to calculate effect sizes for significant actor and partner effects.

To examine the effects of the SHARE intervention on patient and spouse engagement in dyadic coping relative to UMC, we performed a series of ANCOVAs with T_0_ scores as covariates and follow-up T_1_ outcome scores as dependent variables. The main effects tested were treatment group (SHARE or UMC) and role (patient or caregiver). We also examined the treatment group X role interaction.

Finally, to understand whether patient and spouse changes in dyadic coping were associated with changes in their own and each other’s outcomes, a series of APIM analyses were conducted. Change scores for dyadic coping and the outcome measures were calculated by subtracting T_0_ from T_1_ values, and interactions between role and changes in dyadic coping were examined.

## Results

### Sample and Recruitment

#### Study Enrollment and Participation

Sixty-four patient-caregiver dyads were screened and 16 were excluded due to one of the dyad members not being eligible. Of the remaining 48 eligible dyads, 34 (71%) consented. Differences between participants and refusers on demographic and medical characteristics were examined. Results showed that refusers reported significantly greater fatigue on the MDASI (*t* = 2.11, *p* = 0.04). The primary reasons for refusal were either that the patient, caregiver, or both were not interested or that they had too much going on. Four dyads dropped out before returning the baseline survey due to the patient not feeling well-enough to participate or having too much going on. Of the remaining 30 dyads, 15 were randomized to SHARE and 15 to UMC.

#### Participant Characteristics

Patients were mostly male (80%), non-Hispanic White (60%), educated with at least some college credits (73%), middle aged (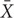 = 58.43, SD = 10.49), and employed full time (60%); 77% had pharynx cancers (63% oropharyngeal, 7% nasopharyngeal, 7% hypopharyngeal) and advanced disease (10% – stage 1, 10% – stage 2, 3% – stage 3, 77% – stage 4A). Twenty-five (83%) of patients were married. Average length of relationship in years was 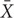 = 28.85 (SD = 12.65; Range = 3–54 years). Spouses were mostly female (77%), non-Hispanic white (63%), educated with at least some college credits (73%), middle aged (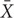 = 58.07, SD = 10.11), and employed full-time (59%).

##### Psychological adjustment

At baseline, no patients and 30% of spouses had PROMIS Depression T-scores >60 (+1SD), indicating high levels of depression. Also at baseline, 27% of patients and 37% of spouses had PROMIS Anxiety T-scores >60, indicating high levels of anxiety. In 17% of dyads, both patient and spouse scored above 60. As **Table 1** shows, partial correlations for patients and spouses for anxiety were significant. Spouses also reported significantly higher depression levels than patients.

**Table 1:** Baseline correlations and descriptive results (*N* = 30 patients and 30 caregivers).

	1	2	3	4	5	6	7	8	9	10	Patients Mean (*SD*)	Spouses Mean (*SD*)	*t*
1. PFSC	**0.16**	0.73^∗∗^	0.38^∗^	0.57^∗∗^	0.11	0.56^∗∗^	−0.45^∗^	−0.23	0.41^∗^	0.12	3.93 (0.70)	3.35 (0.99)	2.81^∗∗^
2. EFSC	0.62^∗∗^	**0.37**^∗^	0.40^∗^	0.80^∗∗^	0.28	0.65^∗∗^	−0.35	0.02	0.22	−0.11	3.27 (1.00)	2.72 (0.99)	2.70^∗∗^
3. PFDC	0.18	−0.03	**0.43**^∗^	0.44^∗^	0.19	0.03	−0.18	0.09	0.48^∗∗^	−0.07	3.62 (0.91)	3.67 (0.95)	−0.27
4. EFDC	0.48^∗∗^	0.49^∗∗^	0.39^∗^	**0.76**^∗∗^	0.08	0.72^∗∗^	−0.36	0.19	0.40	−0.22	3.93 (1.12)	4.10 (1.05)	−1.27
5. NegDC	−0.48^∗∗^	−0.14	−0.12	−0.11	**0.01**	0.20	0.19	0.11	−0.46	0.07	1.57 (0.81)	1.57 (0.72)	0
6. SatDC	0.55^∗∗^	0.40^∗^	0.39^∗^	0.81^∗∗^	0.45	**0.68**^∗∗^	−0.40^∗^	−0.13	0.01	0.01	4.27 (1.05)	3.93 (1.14)	2.16^∗^
7. PROMIS depression raw score	−0.29	−0.10	−0.01	0.36	0.19	0.05	**−0.13**	0.41^∗^	−0.35	−0.05	9.27 (3.31)	12.40 (5.87)	−2.40^∗^
8. PROMIS anxiety raw score	−0.12	0.10	−0.11	0.09	0.15	−0.27	0.53^∗∗^	**0.49^∗∗^**	0.11	−0.68^∗∗^	11.53 (4.17)	13.00 (4.97)	−1.66
9. DAS-7	0.30	0.12	0.22	0.10	−0.32	0.29	−0.10	−0.08	**0.38**^∗^	−0.11	28.80 (4.01)	27.57 (5.28)	1.39
10. Age	0.11	−0.05	−0.25	−0.20	0.07	−0.04	−0.44^∗∗^	−0.28	−0.10	**0.94^∗∗^**	58.43 (10.49)	58.07 (10.11)	0.52

*Partial correlations between patients and spouses are in bold, on the diagonal. Correlations for patients are above the diagonal. Correlations for spouses are below the diagonal. PFSC, problem-focused stress communication; EFSC, emotion-focused stress communication; PFDC, problem-focused dyadic coping; EFDC, emotion-focused dyadic coping; NegDC, negative dyadic coping; SatDC, satisfaction with dyadic coping; DAS-7, short-form Dyadic Adjustment Scale; SD, standard deviation; t, paired samples t-test ^∗^p < 0.05, ^∗∗^p < 0.01.*

##### Marital adjustment

At baseline, 3% of patients and 10% of spouses scored below the DAS-7 cut-off for marital distress. At the 1-month follow-up, 3% of patients and 23% of spouses reported marital distress. Partial correlations for patient and marital adjustment were also significant and in the expected direction (**Table 1**).

### Baseline APIM Analyses

No significant actor or partner interactions between any of the dyadic coping variables and role were found, so the interaction terms were removed and the models rerun. Results are below.

#### Depression

**FIGURE 1:**
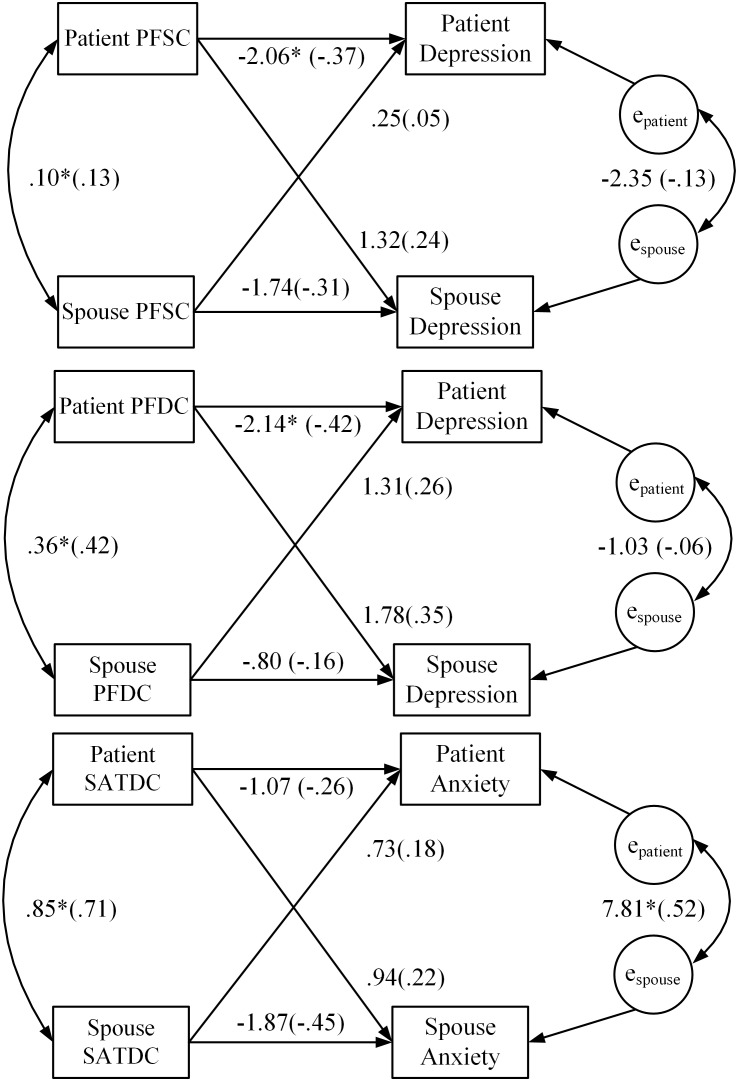
Results of APIM baseline analysis regressing PROMIS depression and anxiety scores on patient and spouse dyadic coping. Model coefficients for the actor and partner effects for both patients and spouses are presented. Standardized coefficients are in parentheses. ^∗^*p* < 0.05; PFSC, problem-focused stress communication; PFDC, problem-focused dyadic coping; SATDC, satisfaction with dyadic coping.

For PFSC, the combined actor effect across patients and spouses was significant (*b* = −1.90, *p* = 0.007) and yielded a medium effect size (*r* = −0.32). However, the combined partner effect was not significant (*b* = 0.79, *p* = 0.33). For PFDC, the combined actor effect across patients and spouses was significant and yielded a small effect size (*r* = −0.29). The combined partner effect was also significant (*b* = 1.55, *p* = 0.04) and yielded a small effect size (*r* = 0.26). For illustrative purposes, **Figure 1** depicts the mixed models coefficients for the actor and partner associations of PFSC and PFDC with depression for patients and spouses separately. By examining the individual coefficients, we discovered that even though the overall actor effects for PFSC and PFDC were significant, the significant association between patient scores on these predictors and patient depression appeared to have been largely driving these effects. Moreover, even though the overall partner effect for PFDC was significant, the individual partner effects (i.e., the effects of patient PFDC on spouse depression and spouse PFDC on patient depression) were not significant.

#### Anxiety

Of all the dyadic coping predictors that we examined, only the combined actor effect of SATDC was significant (*b* = −1.47, *p* = 0.01) and yielded a small effect size (*r* = −0.28). However, the combined partner effect across patients and spouses for SATDC was not significant (*b* = 0.83, *p* = 0.17). **Figure 1** depicts the mixed models coefficients for the actor and partner associations of SATDC and anxiety for patients and spouses. As the figure shows, even though the overall actor effect was significant, the individual actor effects for patients and spouses were not significant.

#### Marital Adjustment

**FIGURE 2:**
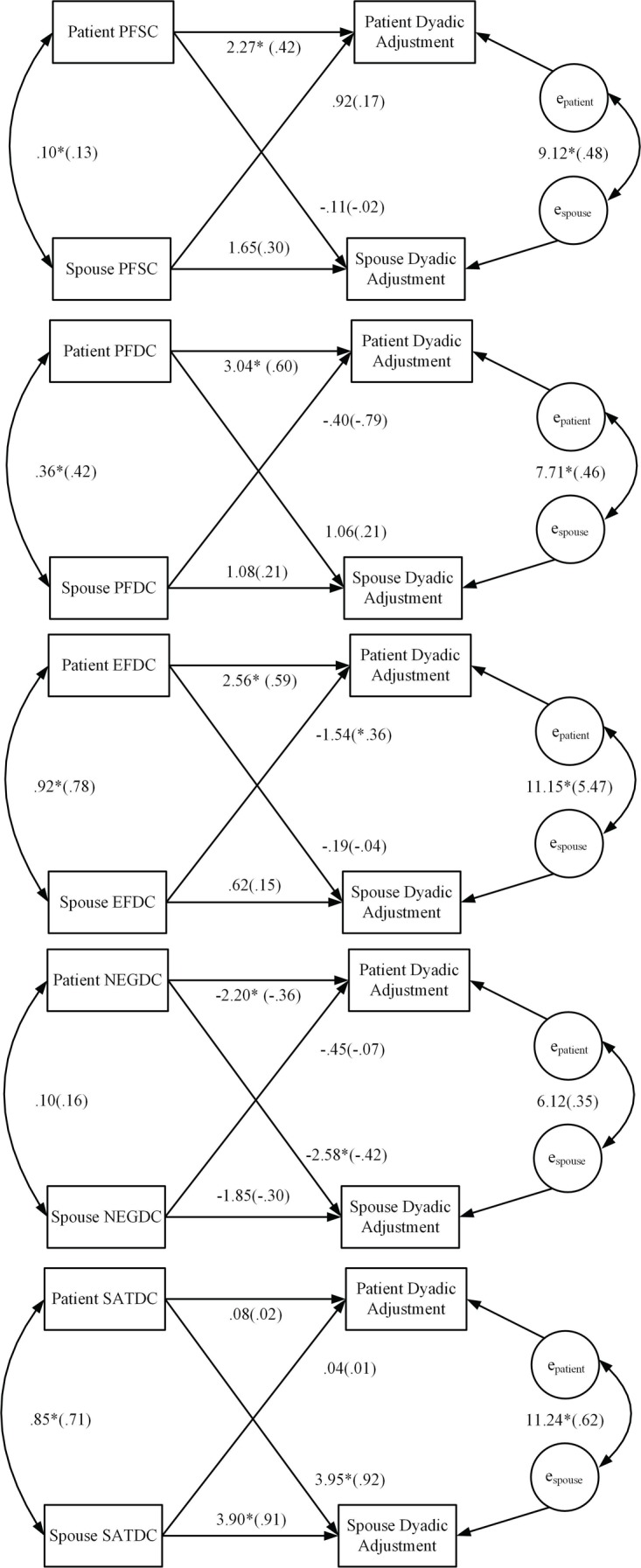
Results of APIM baseline analysis regressing marital (dyadic) adjustment scores on patient and spouse dyadic coping. Model coefficients for the actor and partner effects for both patients and spouses are presented. Standardized coefficients are in parentheses. ^∗^*p* < 0.05, PFSC, problem-focused stress communication; PFDC, problem-focused dyadic coping; EFDC, emotion-focused dyadic coping; NegDC, negative dyadic coping; SatDC, satisfaction with dyadic coping.

Significant combined actor effects across patients and spouses were found for PFSC (*b* = 1.96, *p* = 0.01; *r* = 0.34), PFDC (*b* = 2.06, *p* = 0.001, *r* = 0.38), EFDC (*b* = 1.59, *p* = 0.03, *r* = 0.23), NEGDC (*b* = −2.03, *p* = 0.01, *r* = −0.35), and SATDC (*b* = 1.99, *p* = 0.001, *r* = 0.36), and effect sizes were small to medium. The combined partner effect for NEGDC (*b* = −1.51, *p* = 0.04, *r* = −0.27) was also significant and the effect size was small. **Figure 2** depicts the mixed models coefficients for the actor and partner associations of PFSC, EFDC, PFDC, NEGDC, and SATDC with marital adjustment for patients and spouses separately. By examining the individual coefficients for the actor and partner effects, we discovered that even though the overall actor effects for each of these associations were significant, the coefficients for the associations between patient scores on all of the dyadic coping variables (except SATDC) and patient marital adjustment were significant, and the coefficients for the associations between all of the spouse dyadic coping variables and spouse marital adjustment were not significant (except SATDC). For NEGDC, the overall partner effect was significant but examination of the individual coefficients for patients and spouses showed that only patient engagement in negative dyadic coping had an adverse effect on spouse marital adjustment. Finally, even though the overall partner effect for SATDC was not significant, patient SATDC was significantly positively associated with spouse marital adjustment.

### Treatment Effects

Means for the dyadic coping variables for patients and spouses by treatment group at baseline (T_0_) and follow-up (T_1_) are in **Table 2**. Results of the ANCOVAs for dyadic coping at T_0_ and T_1_ are in **Table 3**. No significant main effects for role or the Group X Role interaction were found. However, at T_1_ there was a significant difference on PFSC (*p* < 0.001), with the SHARE group having higher mean scores (more PFSC) than the UMC group. The effect size for this difference was Cohen’s *d* = 1.14 (95% CI = 0.60 to 1.69), which is a large effect (Cohen, 1988). Also at T_1_, significant differences were found on PFDC (*p* = 0.02) and SATDC (*p* = 0.001) with the SHARE group having higher mean scores than the UMC group. Effect sizes were *d* = 0.64 (95% CI = 0.07 to 1.10) for PFDC, which is a medium effect, and *d* = 1.03 (95% CI = 0.49 to 1.57) for SATDC, which is a large effect. Finally, at T_1_ there was a significant difference in NEGDC (*p* = 0.02), with the SHARE group having lower mean scores that the UMC group. The effect size was *d* = −0.68 (95% CI = −1.21 to −0.16), which is a medium effect.

**Table 2:** Baseline and follow-up means and SDs for stress communication and dyadic coping for patients and spouses in the SHARE intervention and UMC.

	Patients				Spouses			
	SHARE		UMC		SHARE		UMC	
	Baseline M (*SD*)	Follow-up M (*SD*)	Baseline M (*SD*)	Follow-up M (*SD*)	Baseline M (*SD*)	Follow-up M (*SD*)	Baseline M (*SD*)	Follow-up M (*SD*)
PFSC	3.93 (0.73)	4.27 (0.90)	3.93 (0.70)	3.53 (1.16)	3.30 (1.18)	3.83 (1.04)	3.40 (0.81)	2.93 (0.90)
EFSC	3.50 (0.73)	3.57 (0.84)	3.03 (1.20)	3.07 (1.13)	2.90 (0.93)	3.27 (0.88)	2.53 (1.04)	2.47 (1.04)
PFDC	3.77 (1.00)	3.73 (1.02)	3.47 (0.81)	3.00 (0.91)	3.90 (0.85)	4.13 (0.81)	3.43 (1.02)	3.03 (1.06)
EFDC	4.27 (0.73)	4.38 (0.64)	3.49 (1.33)	3.64 (1.14)	4.44 (0.66)	4.49 (0.79)	3.76 (1.26)	3.58 (1.22)
NEGDC	1.73 (1.02)	1.80 (1.05)	1.40 (0.51)	1.97 (0.77)	1.60 (0.83)	1.47 (0.64)	1.53 (0.61)	2.17 (1.21)
SATDC	4.47 (0.64)	4.87 (0.52)	4.07 (1.34)	3.87 (1.30)	3.93 (1.03)	4.27 (0.88)	3.93 (1.28)	3.60 (1.18)

*UMC, usual medical care; M, mean; SD, standard deviation; PFSC, problem-focused stress communication; EFSC, emotion-focused stress communication; PFDC, problem-focused dyadic coping; EFDC, emotion-focused dyadic coping; NegDC, negative dyadic coping; SatDC, satisfaction with dyadic coping. Scores for each of the dyadic coping sub-scales range from 1 to 5, with higher scores indicating more frequent dyadic coping.*

**Table 3:** ANCOVA results for dyadic coping at baseline (T_0_) and at 1-month follow-up (T_1_).

	Treatment group		
Measure at T_1_	*F*-value	*p*-value	Least square means
PFSC	16.29	<0.0001	UMC = 3.20, SHARE = 4.08
EFSC	n.s.	n.s.	–
PFDC	6.29	0.02	UMC = 3.20; SHARE = 3.75
EFDC	n.s.	n.s.	–
NEGDC	5.60	0.02	UMC = 2.16; SHARE = 1.54
SATDC	13.38	0.001	UMC = 3.81; SHARE = 4.49

*UMC, usual medical care; n.s., not significant; PFSC, problem-focused stress communication; EFSC, emotion-focused stress communication; PFDC, problem-focused dyadic coping; EFDC, emotion-focused dyadic coping; NegDC, negative dyadic coping; SatDC, satisfaction with dyadic coping.*

### APIM Change Score Analysis

#### Depression

**FIGURE 3:**
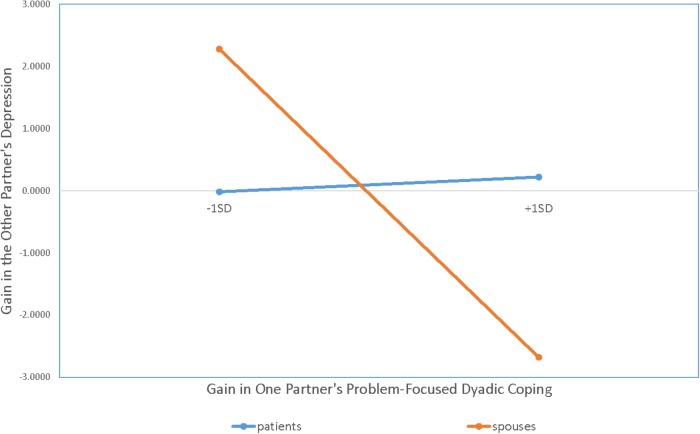
Results of APIM analysis regressing one partner’s gain scores for depression on the other partner’s gain scores from problem-focused dyadic coping. Gain scores for depression were calculated by subtracting T_0_ from T_1_ values on PROMIS depression raw scores.

As **Figure 3** shows, the interaction between role and gains in a partner’s PFSC was significant (*b* = 1.53; *t* = 2.80, *p* = 0.01). Tests of the simple slopes showed that gains in spouses’ PFSC did not significantly affect patients’ depression (*b* = 0.13; *z* = 0.04, *p* = n.s.), but gains in patients’ PFSC resulted in significant reductions in spouses’ depression (*b* = −2.92; *z* = 3.49, *p* = 0.001). A significant main effect was also found for actors’ PFDC (*b* = −1.48; *t* = −2.10, *p* = 0.04).

#### Anxiety

Although none of the role main effects or interactions were significant, significant main effects were found for gains in actors’ PFDC (*b* = −1.77; *t* = −2.69, *p* = 0.01) and SATDC (*b* = −2.14; *t* = −2.80, *p* = 0.01) meaning that improvements in both of these dyadic coping behaviors were associated with reductions in anxiety for both patients and spouses.

#### Marital Adjustment

Although none of the role main effects or interactions were significant, the main effect for gains in actors’ SATDC was significant (*b* = 1.22; *t* = 2.04, *p* = 0.05). In addition, main effects for gains in partners’ PFSC (*b* = 0.80; *t* = 1.70, *p* < 0.10) and partners’ NEGDC (*b* = −0.92; *t* = −1.94, *p* = 0.06) were marginally significant. Thus, there was a trend for patients and spouses to report improvements in marital adjustment when their partners engaged in more PFSC and less NEGDC.

## Discussion

Even though RT for HNC can be stressful for both members of the couple, patients and spouses still find ways to support and care for each other during this emotionally and physically taxing time. Caring for and providing support to a partner who is under stress when one is already experiencing stress of his or her own can be a challenging undertaking that requires coping efforts that address each partner’s well-being as well as the well-being of the relationship. With these points in mind, this study evaluated the effects of dyadic coping on both partner’s psychological and marital adjustment and whether the SHARE intervention resulted in meaningful changes in patient and spouse dyadic coping relative to UMC. Providing partial support for our hypotheses, we found that engaging in some dyadic coping strategies (i.e., PFSC, PFDC, SATDC) was related to psychological and marital adjustment for both partners, whereas engagement in other dyadic coping strategies (i.e., EFDC, NEGDC) was only related to marital adjustment. We also found moderate to large effect sizes for the impact of SHARE relative to UMC on PFSC, PFDC, NEGDC, and SATDC. Finally, we found that increases in one’s PFDC from baseline to the one-month follow-up were consistently associated with improvements in psychological and marital adjustment but that increases in a partner’s PFSC were only associated with improvements in spouses’ depression. Improvements in other dyadic coping strategies (i.e., EFSC, EFDC) were minimal and did not demonstrate significant effects on psychological adjustment, although increases in EFDC were significantly associated with increases in marital adjustment. Taken together, these findings provide important information for future couple-based interventions in HNC.

It is notable that few significant partner effects were found for the baseline APIM analyses that were conducted – and in most cases, the significant actor effects that were found were driven by significant associations for the patient only. When dealing with self-report data, partner effects are often smaller in magnitude (Ackerman et al., 2011). Thus, the small sample size in this study may have made it more difficult to detect such effects. That said, the partner effects that were significant were notable. With regard to depression, both patients and spouses reported lower depression levels when they engaged in PFDC and when their partners engaged in PFDC. Thus, PFDC may be an important target for future couple-based interventions aimed at alleviating patient and partner distress. With regard to marital adjustment, only the partner effect for NEGDC was significant. The fact that none of the partner effects for positive dyadic coping strategies were significant may be consistent with the notion that “bad is stronger than good” (Baumeister et al., 2001). Indeed, studies of marital relationships have consistently shown that the presence or absence of negative behaviors is more strongly related to the quality of couples’ relationships than the presence or absence of positive behaviors (Krokoff et al., 1989; Gottman, 1991, 1994). Gottman (1994) even proposed that in order for a relationship to succeed, positive couple interactions should outnumber the negative ones by at least five to one. In this study, the ratio of positive to negative dyadic coping was approximately 2:1 and thus may not have been strong enough to impact partners’ marital adjustment.

With regard to the finding that more significant associations between dyadic coping and marital and psychological adjustment were found for patients, it is important to point out that tests of all of the dyadic coping by role (i.e., patient or spouse) interactions were not significant. Thus, even though examination of the individual patient and spouse coefficients showed mostly significant associations for patients, we cannot assume that role differences exist. It is possible that the small sample size made it more difficult to detect significant differences in the effects of dyadic coping based on role. Indeed, the published literature supports the idea of role differences. For example, it is possible that by virtue of the illness situation, patients are in more acute need and thus more likely to communicate their need for support to their partners (Badr et al., 2010). Given their caregiving role, spouses in turn may be more likely to provide them with support (Glasdam et al., 1996). Patients may also feel that they are contributing to the relationship and supporting their partner when they engage in PFDC, and this in turn may have positive benefits for their psychological and marital adjustment. Our future work will thus explore whether possible gender and role differences exist with regard to the effects of dyadic coping in a larger sample with sufficient power to simultaneously test for these effects. On a related note, although spouses of cancer patients may be more likely to shield their partners from their own needs and concerns, research has shown that their marital adjustment does benefit from engaging in common positive dyadic coping (Badr et al., 2010), which involves joint efforts to manage the shared stress of the couple (Bodenmann, 2005). We did not assess common dyadic coping in this study, but our future work will examine whether dyadic coping interventions that teach HNC patients to solicit spousal support and engage in PFDC and that teach patients and spouses ways to work together as a team to jointly manage their shared stress are beneficial for both members of the couple.

Another notable finding was that the actor/partner effects of EFSC and EFDC on psychological adjustment were not significant despite the fact that the disclosure of feelings and concerns is a topic that has received considerable research attention and is commonly advocated in couple-based interventions (for a review, see Badr, 2017). The idea that couples should talk about feelings is grounded in social cognitive models that posit that stressful events like cancer are a threat because they challenge existing schemas about the self and relationships (Janoff-Bulman, 1989; McLean et al., 2011). From this perspective, adaptation involves actively assimilating illness into these schemas through acceptance, reappraisal, and disclosure to a supportive partner (Lepore and Revenson, 2007; Kershaw et al., 2008). In the context of HNC, however, this can be challenging because patients (and spouses) are going to the hospital daily for RT and may become so overwhelmed and busy dealing with the day-to-day management of the illness in addition to their daily lives that they may not have the time or energy to process emotions and talk about feelings. Thus, discussions about practical support that is needed or that focus on problem-solving may be more beneficial during this acutely stressful period than discussions about emotions. Discussions about emotions may still be beneficial for HNC couples dealing with the long-term sequelae of RT and struggling to return to a normal life, or those dealing with a cancer recurrence or end-of-life, but more research is needed to follow couples for a longer period post-treatment to determine if EFSC and EFDC have any beneficial effects over time.

A related issue is that even though SHARE evidenced significant positive treatment effects for PFSC, PFDC, NEGDC, and SATDC, it did not significantly impact EFSC or EFDC. In SHARE, skills stress communication and supportive dyadic coping skills were taught through a stress reducing conversation exercise where partners took turns disclosing a topic of concern of their choice and took turns listening to and offering support to one another (Bodenmann and Shantinath, 2004). A review of the audiotapes of this session revealed that in over 50% of these discussions, the “stressor” being discussed was managing the demands of everyday life in addition to the cancer. The remainder of the topics were evenly divided between symptom management issues and wanting partners to acknowledge feelings. Given that the most of the topics of discussion centered around practical or health related topics, the topics themselves may have been more conducive for practicing PFSC and PFDC as opposed to EFSC and EFDC.

With regard to the change score analyses, we found that gains in spouses’ PFSC did not significantly affect patients’ depression, but gains in patients’ PFSC resulted in significant reductions in spouses’ depression. These findings are consistent with our previous work in HNC showing that spouses’ often feel responsible for ensuring that patients adhere to self-management recommendations to control physical symptoms (Badr et al., 2016). Thus, when patients let their spouses know that they need their practical support or advice or ask for their help, spouses may feel more purposeful and in control of an otherwise difficult situation. In addition, although we found that changes in SATDC were associated with changes in marital adjustment, changes in the other dyadic coping strategies did not affect marital adjustment. One reason could be that our sample was highly martially satisfied at baseline and there was not much room for improvement. Future work should examine these associations in more martially distressed couples.

Overall, this study had several strengths. First, to our knowledge, this is the first couple-based dyadic coping intervention in HNC. Second, 40% of patients and 36% of spouses were racial/ethnic minorities, which bolsters generalizability. Other strengths include the rigorous randomized design, and data analytic approach that addressed the dependency among partners. This study also had some limitations. The sample largely comprised patients coping with advanced stage disease (stage 4A), so ability to generalize findings to patients dealing with early stage disease (stages 1–3) is limited. As initial support has now been obtained for SHARE on dyadic coping, it is important to replicate findings with a larger sample size. Given the multiple analyses performed, there was increased potential for error. Since the small sample size likely reduced the parameter estimates, a larger study would allow for examination of gender and role effects as well as the inclusion of additional covariates. A longer follow-up would allow us to examine the maintenance of effects after patients completed the acute recovery period. Given the dyadic nature of the study and the fragility of this patient population, retention over an extended follow-up is likely to be challenging; however, given the fact that SHARE was meant to fortify the couple against the wear and tear of HNC on adjustment, it is important to determine how long intervention effects last, whether booster sessions are needed, and whether receiving the intervention results in decreased healthcare utilization such as unnecessary hospital admissions (i.e., due to poor symptom control at home). Finally, because SHARE had multiple components, it will be important to explore what it is about the intervention that is of benefit and whether the “active ingredients” are the same for patients and spouses.

## Conclusion

Our findings provide evidence that SHARE improved positive dyadic coping and decreased negative dyadic coping for both patients and spouses. They also showed that even during periods of great stress, positive changes in dyadic coping can occur if couples are provided with the appropriate skills training and support; and, that such changes can have a meaningful impact on both partners. Finally, findings suggest that interventions that target dyadic coping can result in improvements on both the individual psychological and marital adjustment levels. Although findings are preliminary and should be interpreted with caution, more research on ways to integrate dyadic coping interventions into oncology supportive care appears warranted.

## Author Contributions

HB contributed to study conceptualization, data collection, data analysis and interpretation, and manuscript writing. KH, JA, MB, and TW contributed to data collection and manuscript writing.

## Conflict of Interest Statement

The authors declare that the research was conducted in the absence of any commercial or financial relationships that could be construed as a potential conflict of interest.
